# Effect of different noise reduction techniques and template matching parameters on markerless tumor tracking using dual‐energy imaging

**DOI:** 10.1002/acm2.13821

**Published:** 2022-11-09

**Authors:** Mandeep Kaur, Peter Wagstaff, Hassan Mostafavi, Mathias Lehmann, Daniel Morf, Liangjia Zhu, Hyejoo Kang, Michal Walczak, Matthew M. Harkenrider, John C. Roeske

**Affiliations:** ^1^ Department of Radiation Oncology, Stritch School of Medicine, Cardinal Bernardin Cancer Center Loyola University Chicago Maywood Illinois USA; ^2^ Department of Radiation Oncology Loyola University Medical Center Maywood Illinois USA; ^3^ Varian Medical Systems Palo Alto California USA

**Keywords:** dual‐energy imaging, image‐guided radiation therapy, lung tumor tracking

## Abstract

**Purpose:**

To evaluate the impact of various noise reduction algorithms and template matching parameters on the accuracy of markerless tumor tracking (MTT) using dual‐energy (DE) imaging.

**Methods:**

A Varian TrueBeam linear accelerator was used to acquire a series of alternating 60 and 120 kVp images (over a 180° arc) using fast kV switching, on five early‐stage lung cancer patients. Subsequently, DE logarithmic weighted subtraction was performed offline on sequential images to remove bone. Various noise reduction techniques—simple smoothing, anticorrelated noise reduction (ACNR), noise clipping (NC), and NC‐ACNR—were applied to the resultant DE images. Separately, tumor templates were generated from the individual planning CT scans, and band‐pass parameter settings for template matching were varied. Template tracking was performed for each combination of noise reduction techniques and templates (based on band‐pass filter settings). The tracking success rate (TSR), root mean square error (RMSE), and missing frames (percent unable to track) were evaluated against the estimated ground truth, which was obtained using Bayesian inference.

**Results:**

DE‐ACNR, combined with template band‐pass filter settings of *σ*_low_ = 0.4 mm and *σ*_high_ = 1.6 mm resulted in the highest TSR (87.5%), RMSE (1.40 mm), and a reasonable amount of missing frames (3.1%). In comparison to unprocessed DE images, with optimized band‐pass filter settings of *σ*_low_ = 0.6 mm and *σ*_high_ = 1.2 mm, the TSR, RMSE, and missing frames were 85.3%, 1.62 mm, and 2.7%, respectively. Optimized band‐pass filter settings resulted in improved TSR values and a lower missing frame rate for both unprocessed DE and DE‐ACNR as compared to the use previously published band‐pass parameters based on single energy kV images.

**Conclusion:**

Noise reduction strategies combined with the optimal selection of band‐pass filter parameters can improve the accuracy and TSR of MTT for lung tumors when using DE imaging.

## 1 INTRODUCTION

Markerless tumor tracking (MTT) is a technique being considered for the management of lung tumor motion, particularly for stereotactic body radiation therapy (SBRT).^1^, ^2^, ^3^, ^4^, ^5^, ^6^, ^7^ Several studies have evaluated MTT with MV^1^, ^2^ or kV^3^, ^4^, ^5^, ^6^, ^7^ images acquired during treatment. MTT using kV imaging relies on the tumor being consistently visible on the image projections.^5^ However, visualizing the tumor is challenging when it is obstructed by a high‐density object, such as bone.^5^ For these cases, various techniques have been investigated to increase the visibility of lung tumors.^4^, ^6^, ^7^, ^8^, ^9^, ^10^ One of these approaches is dual‐energy (DE) subtraction imaging that improves the contrast of lung tumors by removing bone and has been shown to increase the accuracy of MTT.^8^, ^9^, ^10^, ^11^, ^12^, ^13^ However, this improved tumor contrast comes at the cost of increased image noise,^13^ which may decrease the accuracy of MTT. To regain the benefits of bone removal, several noise reduction techniques have been investigated for DE imaging primarily in the diagnostic setting.^14^, ^15^, ^16^, ^17^, ^18^ However, to our knowledge, the impact of DE noise reduction techniques has not been thoroughly studied in the context of MTT.

Although several tracking approaches are being considered for MTT, we and others have been investigating the use of template tracking algorithms.^6^, ^9^, ^10^, ^11^ Template tracking uses 2‐D templates of the tumor generated from the planning CT scan at the corresponding imaging angle. The template is scanned within a defined search window and the normalized cross‐correlation (NCC) is calculated between template and image. The tracked location is the one that maximizes the NCC. To improve tracking performance, a band‐pass filter is often applied to the template and image.^11^ The optimal selection of band‐pass parameters is important in maximizing the success of template tracking.^19^, ^20^ For example, if the *σ* values associated with the band‐pass filter are too low, too much detail is preserved, and very few matched locations are found.^20^ However, if the *σ* values are too high, there is little detail remaining in the images and templates, resulting in a low probability of a match.^20^ A recent study optimized the band‐pass filter parameters on single energy (SE) kV images for template tracking.^20^ They determined that the combination of *σ*_low_ = 0.6 mm and *σ*_high_ = 2.4 mm worked best for most patients to improve the tracking accuracy on SE images.

Due to the inherent differences between DE and SE images, as well as the additional noise processing techniques that may be applied to DE images, it is not clear that the band‐pass parameters determined by Hazelaar^20^ will be optimal for MTT using DE images. Thus, the goal of this study is to evaluate various DE noise reduction techniques, along with template tracking parameters, to determine the optimal combination that maximizes the success of MTT with DE imaging.

## 2 MATERIALS AND METHODS

### 2.1 Patient data and dual‐energy subtraction

Five early‐stage lung cancer patients (Table 1) treated with SBRT were imaged as part of an IRB‐approved study. Prior to treatment, all patients underwent a 4D‐CT simulation and treatment planning according to our institutional protocol.^21^ Patients were simulated in the supine position and immobilized using an alpha cradle indexed to the treatment table. Using the 4D‐CT, an internal target volume was generated by contouring (Eclipse, Varian Medical Systems, Palo Alto, CA) the gross tumor volume on the free‐breathing, 0%‐phase, 50%‐phase, and the maximum‐intensity‐projection series. Treatment planning was performed following our standard methods.^21^ All plans used volumetric modulated arc therapy and were optimized to meet our SBRT dose constraints. Typically, 2–3 arcs were used per fraction with an overall prescription dose of 5000–6000 cGy delivered in 3–5 fractions to the planning target volume.

**TABLE 1:** Summary of tumor location and dimensions for each patient

			Maximum dimension (mm)		
Patient	Tumor location	GTV (cc)	AP	LR	SI
1	RUL	1.9	15	18	14
2	RUL	7.3	22	24	26
3	LUL	3.5	18	22	17
4	RUL	14.4	28	26	34
5	RUL	8.3	39	26	20

Abbreviations: AP, anterior–posterior; GTV, gross tumor volume; LR, left‐right; LUL, left upper lobe; RUL, right upper lobe; SI, superior–inferior.

At the completion of one of the treatment fractions, for each patient, a series of alternating projections were obtained at 60 and 120 kVp over a 180° arc using the fast kV switching capabilities of the onboard imager in Developer Mode (TrueBeam, Varian Medical Systems, Palo Alto, CA).^10^ Images were acquired at 15 frames/s producing a total of 450 high/low‐energy images. The mA setting for the images was adjusted to minimize the difference in air exposure between the 60 kVp (60 mA, 20 ms) and 120 kVp (15 mA, 20 ms) acquisitions.^9^, ^10^, ^11^ To create the DE images, weighted logarithmic subtraction (WLS) was performed offline on paired 60/120 kVp images to reduce bone present in the resultant soft‐tissue image.^9^, ^10^, ^11^

This study required the production of both soft‐tissue DE images (*I*_DEST_), as well as bone DE images (*I*_DEB_) for use with subsequent noise reduction algorithms. We performed WLS to generate *I*_DEST_ and *I*_DEB_ as follows^22^:

(1) $$I_{\mathrm{DEST}}=\ln I_{H}-w_{\mathrm{ST}}\ln I_{L}$$

(2) $$I_{\mathrm{DEB}}=-\ln I_{H}+w_{B}\ln I_{L}$$

where *I_H_* and *I_L_* are the intensities of individual pixels on the high‐ and low‐energy projections, respectively. The weighting factors, *w_ST_* and *w_B_*, were used to produce *I*_DEST_ and *I*_DEB_ images and were empirically determined to be 0.42 and 0.70, respectively. Various noise reduction techniques were applied to these DE images using software written in MATLAB R2021a (MathWorks, Natick, MA, USA) and are discussed in the following sections.

### 2.2 Simple smoothing (SS) of the high‐energy image

The high‐energy image was smoothed using a median filter prior to DE image processing.^14^, ^15^ The rationale is that most of the noise in the resultant DE image arises from the high‐energy image component.^15^ Additionally, the unfiltered low‐energy projection (*I_L_*) preserves high spatial‐frequency information.^14^ In the present study, we used a 3 × 3 median filter on the high‐energy image (*I_H_*) as follows:

(3) $$I_{H}^{{}^{\prime }}=\mathrm{median}\left(I_{H}\right)$$

(4) $$I_{\mathrm{SSF}}=\ln I_{H}^{\prime }-w_{ST}\ln I_{L}$$

where *I'_H_* is the simple smoothed high‐energy image, and *I*_SSF_ is the resultant DE image following simple smoothing (SS).

### 2.3 Anti‐correlated noise reduction (ACNR)

The anticorrelated noise reduction (ACNR) algorithm takes advantage of the fact that the noise is anticorrelated between the DE bone and soft‐tissue images.^16^ Kalender et al. demonstrated that the noise in the complementary image can be reduced by estimating the noise in either the bone or tissue image. To reduce noise in the soft‐tissue image, the ACNR algorithm applies a high‐pass filter (average filter with kernel size of 20 × 20 pixels) to the complementary image, that is, the bone‐only image (*I*_DEB_). This filter effectively removes the bone from the complementary image, leaving the noise behind. The original DE soft‐tissue image (*I*_DEST_) and high‐pass filtered bone image ($I_{\mathrm{DEB}}^{\mathrm{HPF}}$) are then added, weighted by a parameter, *w_n_* ^18^:

(5) $$I_{\mathrm{DEST}}^{\mathrm{ACNR}}=I_{\mathrm{DEST}}+w_{n}I_{\mathrm{DEB}}^{\mathrm{HPF}}$$

The optimal value of *w_n_* was determined empirically to be 0.35.

### 2.4 Noise clipping (NC)

The noise‐clipping (NC) technique, developed by Hinshaw et al., is based on the concept that due to increased photoelectric effect, structures in the low‐energy image (*I_L_*) should have a higher contrast that those in the high‐energy image (*I_H_*).^17^, ^18^ A median filter kernel (17 × 17) was used to estimate the background signal in both images.^18^ The background was then subtracted from individual pixel values in both images, and the contrast of each paired pixel in the resultant images was compared. If the high‐energy pixel contrast was greater than that of the low energy, the increased contrast was attributed to noise, and its value was clipped. That is, the high‐energy pixel value is adjusted to match the contrast of the low‐energy pixel. The algorithm is implemented on a pixel‐by‐pixel basis:

(6) $$\mathrm{Th}\left(\mathrm{Threshold}\right)=I_{L}-\mathrm{median}\left(I_{L}\right)$$

(7) $$\mathrm{if}I_{H}-\mathrm{median}\left(I_{H}\right)>\mathrm{Th}$$

then

(8) $$I_{H}=\mathrm{Th}+\mathrm{median}\left(I_{H}\right)$$

**FIGURE 1:**
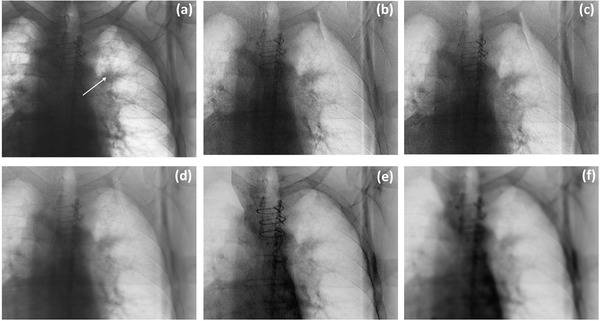
(a) Single energy (SE), (b) dual energy (DE) unprocessed, (c) DE‐simple smoothing (SS), (d) DE‐anticorrelated noise reduction (ACNR), (e) DE‐noise clipping (NC), and (f) DE‐NC‐ACNR images for patient 5 obtained at an anterior imaging angle. The arrow on (a) indicates the position of the tumor.

The resultant noise‐clipped high‐energy image was used to produce a DE image using WLS, defined in Equation (1). Furthermore, we also performed ACNR using the noise‐clipped high‐energy images as discussed in the previous section. An example of the unprocessed and noise‐filtered DE images for a representative patient is shown in Figure 1.

### 2.5 Template tracking

To determine the position of the tumor, template matching was performed using nonclinical offline research software (RapidTrack Offline [RTO.3.0.3.0] Varian Medical Systems, Palo Alto, CA, USA).^23^ Tracking is a two‐step process consisting of template generation, followed by the tracking itself. First, 2D templates of the tumor were derived from the 50% phase of the 4D‐planning CT scan for each individual patient. These templates were generated for every 1° of gantry rotation. Templates and images were preprocessed using a band‐pass filter software that was implemented by subtracting a high‐pass Gaussian‐shaped kernel (*σ*_high_) from a low‐pass Gaussian‐shaped kernel (*σ*_low_). To optimize the band‐pass filter settings for the present study, 20 different combinations of *σ*_low_ and *σ*_high_ values were used. *σ*_low_ was varied from 0.2 to 0.8 mm with a step size of 0.2 mm, whereas *σ*_high_ values were obtained by multiplying *σ*_low_ by 2–5, similar to work done by Hazelaar.^19^, ^20^ These templates were used with each combination of DE images (unprocessed, DE‐SS, DE‐ACNR, DE‐NC, and DE‐NC‐ACNR) to evaluate the accuracy of MTT. Following template generation, the tracking algorithm identifies the location of the tumor by scanning the created template across a search region (dependent on the size of the tumor) positioned on the expected position of the tumor for each kV projection. At each location, the NCC is calculated, and the location where the NCC is maximized is considered the tracked position.^23^

### 2.6 Ground‐truth estimation and tracking metrics

In clinical images, the ground‐truth (GT) for MTT is not known. Hence, estimated GT positions were determined using Bayesian inference based on the Kalman filter (KF) with constant acceleration.^24^, ^25^, ^26^ The KF provides an effective estimator for the position of a dynamical system in the presence of measurement error. As such, it is a statistical inference approach to estimate the true position of an object by combining the predicted and measured positions. Previous studies have used the KF approach to estimate the 3D position of tumors from measured data.^24^, ^25^ In the case of prostate cancer, Nguyen et al. demonstrated that the KF can estimate the true prostate position with submillimeter precision and accuracy.^25^ In the current study, the KF is used to estimate GT through an iterative two‐step process of prediction and correction.^24^, ^25^ The variables associated with the KF equations are defined in Table 2.

**TABLE 2:** List of variables and their description used in the Kalman filter (KF) to estimate the ground‐truth (GT) for tumor motion^24^, ^25^

Variable	Description of Kalman parameters
*k*	Current image frame
*x_k_*	Estimated 2D position of the target in the current frame
*F*	State transition matrix of the system between frames
*u*	Input control vector defining the system dynamics
*G*	System input matrix applied to control *u*
*Q*	Process noise covariance matrix for the current frame *k*
*z_k_*	Measurement vector of the 2D position of the tumor in the current frame
*P_k_*	Predicted error covariance matrix for the current frame
*H_k_*	Observation matrix
*K_k_*	Kalman gain, representing the weighting parameter for how much of the measurement is used to correct the prediction
*R_k_*	Covariance of the observation noise for the current frame
*I*	Identity matrix

Briefly, the prediction step estimates the tumor position prior to measurement using a state transition model.^24^ The predicted vector position $x_{k_{p}}$ in the *k*th frame is based on the information of tumor motion from the previous frame (*k*−1) and is given by

(9) $$x_{k_{p}}=Fx_{k-1}+Gu$$

Similarly, the measurement error covariance matrix, *P_kp_*, is estimated based on the previous error *P_k_*_−1_ as given by

(10) $$P_{k_{p}}=FP_{k-1}F^{T}+Q$$

Using the current measurement, *z_k_*, the estimated position *x_k_* (our estimated GT) is given by

(11) $$x_{k}=x_{k_{p}}+K_{k}\left[z_{k}-H_{K}\left(x_{k_{p}}\right)\right]$$

*P_k_* is calculated for future prediction and update:

(12) $$P_{k}=\left(I-K_{k}H_{k}\right)P_{k_{p}}$$

The Kalman gain factor, *K_k_*, which is used to correct the prediction, is given by

(13) $$K_{k}=\frac{P_{k_{p}}H_{k}^{T}}{H_{k}P_{k_{p}}H_{k}^{T}+R_{k}}$$

An additional theoretical foundation can be obtained in the original paper by Kalman.^26^

Using the estimated GT from the KF, a quantitative analysis was performed based on tracking success rate (TSR), root mean square error (RMSE), and the percentage of missing frames (number of frames that were not tracked by the MTT software). The TSR is based on the difference between the actual and tracked locations. A successful tracking event on a particular image frame is defined as a difference between the tracked location and estimated GT positions of <2 mm. RMSE was calculated by the square root of the square of the difference between the actual tracked location and GT of the target, divided by the number of frames for each patient as given by

(14) $$\mathrm{RMSE}=\sqrt{\frac{\sum_{i=1}^{N_{\mathrm{tr}}}{\left(x_{i}-x_{t}\right)}^{2}}{N_{\mathrm{tr}}}}$$

where *x_i_* and *x_t_* are actual tracked location and estimated GT, respectively, and *N*_tr_ is the total number of tracked frames per patient.

## 3 RESULTS AND DISCUSSION

### 3.1 Effect of noise reduction techniques on DE images

**FIGURE 2:**
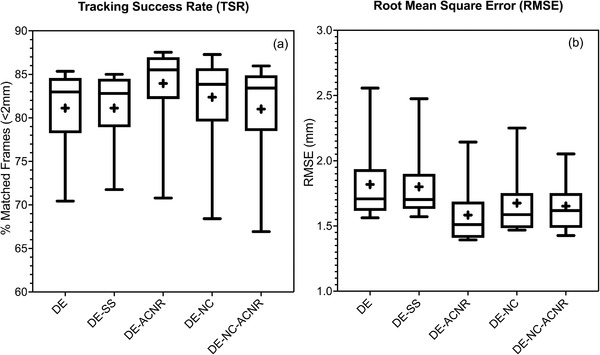
(a) Tracking success rate (TSR) and (b) root mean square error (RMSE) of dual‐energy (DE)‐filtered images using different noise reduction techniques and compared with DE‐unfiltered. (+,− signs are showing mean and median values, respectively; whiskers outside the box are showing the minimum and maximum values for TSR and RMSE, respectively.)

In total, the data from five patients resulted in 1140 DE images that were analyzed for the unprocessed and noise‐filtered DE images for a total of 5700 images. Figure 2 shows a box‐and‐whiskers plot of the TSR (Figure 2a) and RMSE (Figure 2b) for each image processing technique and all template combinations. As a baseline, unprocessed DE images resulted in median TSR = 82.9% and RMSE = 1.71 mm. In comparison, DE‐SS had TSR and RMSE values of 82.8% and 1.70 mm, respectively, which were not statistically different from the unprocessed DE images (Wilcoxon matched pair test). DE‐ACNR resulted in statistically larger TSR values (median = 85.5%, *p* < 0.01) and lower RMSE (median = 1.51 mm, *p* < 0.01) compared with the unprocessed DE images. The application of NC resulted in a median TSR = 83.9% (*p* = 0.02, relative to DE) and RMSE = 1.59 mm (*p* < 0.01, relative to DE) was statistically significant. DE‐NC‐ACNR resulted in median TSR = 83.4% (*p* = 0.87, relative to DE), which was not statistically significant, but the RMSE = 1.62 mm (*p* = 0.012, relative to DE) was statistically significant. Based on these results, further analysis was performed on both DE and DE‐ACNR images, as DE‐ACNR provided the most significant improvements both in TSR and RMSE.

**FIGURE 3:**
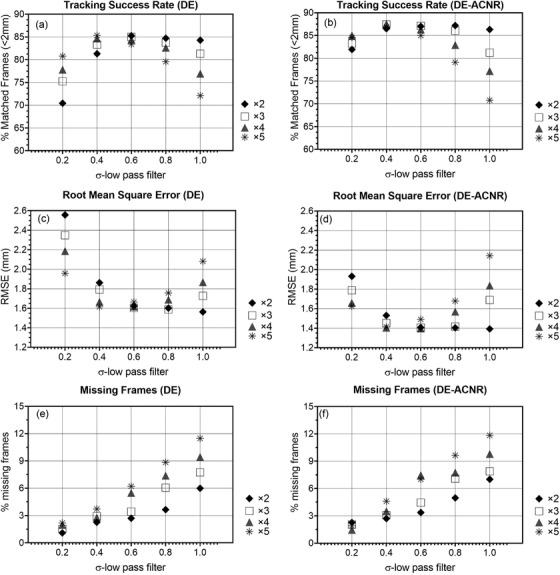
Tracking success rate (TSR) (a) + (b), root mean square error (RMSE) (c) + (d), and missing frames (e) + (f) for dual‐energy (DE) and DE‐anticorrelated noise reduction (ACNR) averaged over five patients' data, for template matching using different combinations of *σ*_low_ and *σ*_high_ for the band‐pass filter. The horizontal axis lists the *σ*_low_ values, whereas *σ*_high_ values are multiples of 2–5 times *σ*_low_ values and are indicated by the respective symbol.

The comparison of DE and DE‐ACNR for different band‐pass filter settings is shown in Figure 3. For both DE and DE‐ACNR, the TSR peaks at *σ*_low_ values ranging from 0.4 to 0.6 mm and rapidly decreases for higher values. Of interest, DE achieves a maximum TSR at *σ*_low_ = 0.6 mm and *σ*_high_ = 1.2 mm (TSR = 85.3%), whereas DE‐ACNR has a maximum TSR at *σ*_low_ = 0.4 mm and *σ*_high_ = 1.6 mm (TSR = 87.5%). In examining RMSE, for both DE and DE‐ACNR, it has minimum values from *σ*_low_ = 0.4–1.0 mm with the lowest RMSE obtained with a smaller value of *σ*_high_. Of note, the minimum RMSE for DE is approximately 1.62 mm, whereas the minimum value for DE‐ACNR is 1.40 mm. The final analysis shows the percent missing frames where the algorithm was unable to track the tumor. From Figure 3, the percent missing frames increase nearly linearly as a function of *σ*_low_ for both DE and DE‐ACNR. Although the lowest percentage of missing frames occurs at *σ*_low_ = 0.2 mm, this occurs at the expense of lower TSR and higher RMSE.

### 3.2 Template matching optimization: band‐pass filter settings

Based on our three factors, TSR, RMSE, and the percent missing frames, we seek to determine the best band‐pass parameter settings for unprocessed DE and DE‐ACNR. Table 3 shows the optimal combination of parameters along with the results using the parameters that were optimized for SE imaging. From this analysis, there is an advantage of using DE‐ACNR versus unprocessed DE due to an increase in TSR and a reduction in RMSE. In both cases, the percent of missing frames is comparable. In comparison to the SE optimized values from Hazelaar et al.,^20^ using DE‐optimized band‐pass parameters provides an increase in TSR and comparable RMSE values. However, the most significant advantage of using DE‐optimized parameters is an ∼50% relative reduction in the number of missing frames where the software is unable to track the tumor. A low missing frame rate is essential for the eventual clinical implementation of such an approach.

**TABLE 3:** Summary of optimal tracking parameters by technique

Technique	Band‐pass filter parameters	Tracking success rate (%)	RMSE (mm)	Missing frames (%)
DE (unprocessed)	*σ*_low_ = 0.6 mm, *σ*_high_ = 1.2 mm	85.3	1.62	2.7
	*σ*_low_ = 0.6 mm, *σ*_high_ = 2.4 mm [20]	84.4	1.62	5.5
DE‐ACNR	*σ*_low_ = 0.4 mm, *σ*_high_ = 1.6 mm	87.5	1.40	3.1
	*σ*_low_ = 0.6 mm, *σ*_high_ = 2.4 mm [20]	86.3	1.40	7.5

Abbreviations: ACNR, anticorrelated noise reduction; DE, dual energy; RMSE, root mean square error.

Although motion tracking with DE images is currently not clinically available, the use of optimized template parameters and application of ACNR to DE images would fit seamlessly into a future clinical workflow. Templates for motion tracking would be generated for the planning CT scan prior to patient treatment and motion tracking. At this stage, the optimal band‐pass filter settings (*σ*_low_ = 0.4 mm and *σ*_high_ = 1.6 mm) would be utilized. During treatment and image acquisition, soft‐tissue and bone DE images would be produced using logarithmic subtraction with appropriate weighting factors. The high‐pass filtered bone DE image is combined with the soft‐tissue DE image using the optimized noise cancelation weighting factor as described in Section 2.3 to produce DE‐ACNR images. All these calculations are matrix based and hence are computationally efficient, allowing them to be performed in near real‐time. Motion tracking, using the previously calculated templates would then be performed on the DE‐ACNR images.

To our knowledge, this is the first study to evaluate DE noise reduction techniques and to determine the optimal band‐pass parameters for template‐based motion tracking. However, there are a number of limitations. Among these, the most important are the study‐optimized parameters based on a small series of patients. However, as shown in the TSR and RMSE plots, there are clear maxima and minima values, respectively. The inclusion of additional patients is not expected to change the overall results; however, it may impact the absolute values (i.e., TSR and RMSE). Additionally, as this study uses patient data, GT was not directly assessable and had to be estimated using the KF. Although the KF has been shown to produce the best estimate of the underlying signal from noisy data,^24^, ^25^, ^26^ it nonetheless does not provide an absolute measurement of GT as would be obtained from a phantom. However, most phantoms do not have realistic anatomy and tumor geometries, as well as respiratory and cardiac motion. Hence, we believe the inclusion of these factors outweigh the use of an estimated GT. Future studies will consider of the use of deep learning‐based tracking algorithms, and how DE image quality may impact the accuracy of tumor tracking using these approaches.

## AUTHOR CONTRIBUTIONS

All listed authors contributed to the work and to writing the article.

## CONFLICT OF INTEREST

HM, ML, DM, LZ and MW were or are employed by Varian Medical Systems.

## References

[1] Serpa M , Baier K , Cremers F , Guckenberger M , Meyer J . Suitability of markerless EPID tracking for tumor position verification in gated radiotherapy. Med Phys. 2014;41(3):031702. 24593706 10.1118/1.4863597

[2] Rottmann J , Keall P , Berbeco R . Markerless EPID image‐guided dynamic multi‐leaf collimator tracking for lung tumors. Med Phys. 2013;58(12):4195‐4204. 10.1088/0031-9155/58/12/4195 PMC4182919 23715431

[3] Lewis JH , Ruijiang L , Watkins WT , et al. Markerless lung tumor tracking and trajectory reconstruction using rotational cone‐beam projections: a feasibility study. Phys Med Biol. 2010;55(9):2505‐2522. 20393232 10.1088/0031-9155/55/9/006

[4] Yang Y , Zhong Z , Guo X , et al. A novel markerless technique to evaluate daily lung tumor motion based on conventional cone‐beam CT projection data. Int J Radiat Oncol Biol Phys. 2012;82(5):749‐756. 22330989 10.1016/j.ijrobp.2011.11.035

[5] Van Sörnsen de Koste JR , Dahele M , Mostafavi H . Markerless tracking of small lung tumors for stereotactic radiotherapy. Med Phys. 2015;42(4):1640‐1652. 25832054 10.1118/1.4914401

[6] Rozario T , Bereg S , Yan Y , et al. An accurate algorithm to match imperfectly matched images for lung tumor detection without markers. J Appl Clin Med Phys. 2015;16(3):131‐140. 26103480 10.1120/jacmp.v16i3.5200 PMC5690140

[7] Shieh CC , Keall PJ , Kuncic Z , Huang C , Feain I . Markerless tumor tracking using short kilovoltage imaging arcs for lung image‐guided radiotherapy. Phys Med Biol. 2015;60(24):9437‐9454. 26583772 10.1088/0031-9155/60/24/9437 PMC4833659

[8] Hoggarth MA , Luce J , Syeda F . Dual energy imaging using a clinical on‐board imaging system. Phys Med Biol. 2013;58(12):4331‐4340. 23732651 10.1088/0031-9155/58/12/4331

[9] Patel R , Panfil J , Campana M , et al. Markerless motion tracking of lung tumors using dual‐energy fluoroscopy. Med Phys. 2014;42(1):254‐262. 10.1118/1.4903892 25563265

[10] Haytmyradov M , Mostafavi H , Wang A , et al. Markerless tumor tracking using fast‐kV switching dual‐energy fluoroscopy on benchtop system. Med Phys. 2019;46(7):3235‐3244. 31059124 10.1002/mp.13573 PMC6625841

[11] Roeske JC , Mostafavi H , Haytmyradov M , et al. Characterization of markerless tumor tracking using the on‐board imager of a commercial linear accelerator equipped with fast‐kV switching dual‐energy imaging. Adv Radiat Oncol. 2020;5(5):1006‐1013. 33089019 10.1016/j.adro.2020.01.008 PMC7560565

[12] Mueller M , Poulsen P , Hansen R , et al. The markerless lung target tracking AAPM grand challenge (MATCH) results. Med Phys. 2022;49:1161‐1180. 34913495 10.1002/mp.15418 PMC8828678

[13] Richard S , Siewerdsen JH . Cascaded systems analysis of noise reduction algorithms in dual‐energy imaging. Med Phys. 2008;35(2):586‐601. 18383680 10.1118/1.2826556

[14] Johns PC , Yaffe MJ . Theoretical optimization of dual‐energy X‐ray imaging with application to mammography. Med Phys. 1985;12(3):289‐296. 4010633 10.1118/1.595766

[15] Kappadath SC , Shaw CC . Dual‐energy digital mammography for calcification imaging: noise reduction techniques. Phys Med Biol. 2008;53(19):5421‐5443. 18765887 10.1088/0031-9155/53/19/010 PMC2858626

[16] Kalender WA , Klotz E , Kostaridou E . An algorithm for noise suppression in dual‐energy CT material density images. IEEE Trans Med Imaging. 1998;7(3):218‐224. 10.1109/42.7785 18230472

[17] Hinshaw DA , Dobbins JT . Recent progress in noise reduction and scatter correction in dual‐energy imaging. Proc SPIE. 1995;2432:134‐142.

[18] Warp RJ , Dobbins JT . Quantitative evaluation of noise reduction strategies in dual‐energy imaging. Med Phys. 2003;30(2):190‐198. 12607836 10.1118/1.1538232

[19] Hazelaar C , Dahele M , Mostafavi H , Van Der Weide L , Slotman BJ , Verbakel W . Subsecond and submillimeter resolution positional verification for stereotactic irradiation of spinal lesions. Int J Radiat Oncol Biol Phys. 2016;94(5):1154‐1162. 27026317 10.1016/j.ijrobp.2016.01.006

[20] Hazelaar C , Dahele M , Mostafavi H , Van Der Weide L , Slotman BJ , Verbakel W . Markerless positional verification using template matching and triangulation of kV images acquired during irradiation for lung tumors treated in breath‐hold. Phys Med Biol. 2018;63(11):115005. 29714710 10.1088/1361-6560/aac1a9

[21] Harris AA , Stang K , Small C , et al. Pretreatment factors influencing radiation pneumonitis after stereotactic body radiation therapy in the treatment of lung cancer. Cureus. 2020;12(3):e7462. doi:10.7759/cureus.7462 32351841 PMC7188020

[22] Brody WR , Butt G , Hall A , Macovski A . A method for selective tissue and bone visualization using dual energy scanned projection radiography. Med Phys. 1981;8(3):353‐357. 7033756 10.1118/1.594957

[23] Mostafavi H , Jeung H , Sloutsky A . WE‐A‐134‐07: RapidTrack: a versatile tool for template‐based target tracking during radiotherapy. Med Phys. 2013;40(6):470‐470.

[24] Shieh CC , Caillat V , Dunbar M , et al. A Bayesian approach for three‐dimensional markerless tumor tracking using kV imaging during lung radiotherapy. Phys Med Biol. 2017;62(8):3065‐3080. 28323642 10.1088/1361-6560/aa6393 PMC5729104

[25] Nguyen DT , Keall P , Booth J , Shieh CC , Poulsen P , Brien P . A real‐time IGRT method using a Kalman filter framework to extract 3D positions from 2D projections. Phys Med Biol. 2021;66:214001. 10.1088/1361-6560/ac06e3 34062512

[26] Kalman R . A new approach to linear filtering and prediction problems. J Basic Eng. 1960;82(D):35‐45.

